# Bisebromoamide, an extract from *Lyngbya* species, induces apoptosis through ERK and mTOR inhibitions in renal cancer cells

**DOI:** 10.1002/cam4.53

**Published:** 2013-02-03

**Authors:** Kenjiro Suzuki, Ryuichi Mizuno, Kiyotake Suenaga, Toshiaki Teruya, Nobuyuki Tanaka, Takeo Kosaka, Mototsugu Oya

**Affiliations:** 1 Department of Urology, Keio University School of Medicine35 Shinanomachi, Shinjuku-ku, Tokyo, 160-8582, Japan; 2 Department of Chemistry, Faculty of Science and Technology, Keio UniversityHiyoshi 3-14-1, Kohoku-ku, Yokohama, 223-8522, Japan; 3 Faculty of Education, University of the Ryukyus1 Senbaru, Nishihara, Okinawa, 903-0213, Japan

**Keywords:** Akt, apoptosis, Bisebromoamide, ERK, mTOR, renal cell carcinoma

## Abstract

Advanced renal cell carcinoma (RCC) remains an incurable disease, and newer anticancer drugs are needed. Bisebromoamide, a novel cytotoxic peptide, was isolated from the marine cyanobacterium *Lyngbya* species at our laboratory in 2009. This compound specifically inhibited the phosphorylation of ERK in platelet-derived growth factor-activated normal rat kidney cells. The aim of this study was to evaluate the effect and elucidate the potential mechanism of Bisebromoamide actions on human RCC cells. Two renal cancer cell lines, 769-P and 786-O, were used. The effects of Bisebromoamide were analyzed employing assays for water-soluble Tetrazolium-1 salts. Apoptosis was determined by flow cytometric TUNEL analysis. Cell-cycle distributions were analyzed by flow cytometry using BrdU/propidium iodide (PI) staining. Kinases of the phosphatidylinositol 3-kinase (PI3K)/Akt/mammalian target of Rapamycin (mTOR) pathway and Raf/MEK/ERK pathway were analyzed by Western blotting. After Bisebromoamide treatment for 48 and 72 h, cell viability was significantly decreased in both cell lines at 1 and 10 μmol/L. After treatment with 1 μmol/L Bisebromoamide for 72 h, apoptosis and the increased percentage of cells in the sub-G1 phase were observed in both cell lines. Bisebromoamide inhibited the phosphorylation of ERK and Akt in both cell lines tested. Similar effects were demonstrated for phosphorylation of mTOR and p70 S6. Bisebromoamide is a promising potential agent against RCC due to its ability to inhibit both the Raf/MEK/ERK and PI3K/Akt/mTOR pathways.

## Introduction

Therapeutic options used in patients with advanced renal cell carcinoma (RCC) were limited, but this has changed dramatically in the last few years [1–5]. Previously, traditional chemotherapy, hormonal therapy, or radiation was not effective in the treatment of advanced RCC, and immunotherapy provided only limited benefit. Improvements in our understanding of the molecular basis of RCC have led to the development of targeted agents tailored to inhibiting intracellular signal transduction pathways that drive angiogenesis, tumorigenesis, and progression. Two signaling pathways, namely Raf/MEK/ERK and phosphatidylinositol 3-kinase (PI3K)/Akt/mammalian target of Rapamycin (mTOR) pathways, play central roles in many types of tumor cell proliferation, and can lead to aberrant signaling and uncontrolled proliferative diseases [6]. Therefore, we focused on these two pathways which were known to be upregulated in many types of cancer, including RCC.

BAY 43-9006 targeting Raf, as well as vascular endothelial growth factor (VEGF), and RAD001 and CCI-779 targeting mTOR are currently in widespread clinical use for patients with advanced RCC. Their common biological targets were von Hippel–Lindau (VHL)/hypoxia inducible factor/VEGF pathway to inhibit angiogenesis, and Raf/MEK/ERK and PI3K/Akt/mTOR pathways to inhibit cancer cell proliferation. Although these targeted agents have improved objective response rates and progression-free survival as compared with prior standard of care agents, advanced RCC remains an incurable disease and newer anticancer drugs are needed.

A novel cytotoxic peptide, Bisebromoamide, was isolated from the marine cyanobacterium *Lyngbya* species harvested in Okinawa, Japan, at our laboratory in 2009 [7,8]. This compound specifically inhibited the phosphorylation of ERK in platelet-derived growth factor-activated normal rat kidney cells. As the ERK pathway is upregulated in many types of cancers, we consider this extract from *Lyngbya* species to have the potential to inhibit RCC cell proliferation. We aimed to evaluate the direct antitumor effect and elucidate the potential mechanism of Bisebromoamide actions on human RCC cells.

## Materials and Methods

### Reagents

Bisebromoamide was obtained from marine cyanobacterium *Lyngbya* species collected at Bise in Okinawa. The isolation procedure was described in a previous report [7]. This agent was solubilized in DMSO and stored in the dark at 4°C until use. Rabbit polyclonal antibodies against total ERKs (t-ERKs), phospho-specific ERKs (p-ERKs), phospho-specific p70 S6 kinase (p-p70 S6 kinase) at Thr389 or Thr421/Ser424, phospho-specific mTOR (p-mTOR) at Ser2448 or Ser2481, total MEK (t-MEK), total PDK1 (t-PDK1), total PI3K (t-PI3K), phospho-specific PI3K (p-PI3K), and cleaved caspase-3 were obtained from Cell Signaling Technology (Beverly, MA). Rabbit monoclonal antibodies against total Akt (t-Akt), phospho-specific Akt (p-Akt) at Ser473, total mTOR (t-mTOR), total p70 S6 kinase (t-p70 S6 kinase), phospho-specific MEK (p-MEK), phospho-specific PDK1 (p-PDK1), total epidermal growth factor receptor (t-EGFR), and phospho-specific EGFR (p-EGFR) were also obtained from Cell Signaling Technology. A mouse monoclonal antibody against β-actin was purchased from Sigma (St. Louis, MO).

### Cell lines and cultures

The two renal cancer cell lines, 769-P and 786-O (purchased from American Type Culture Collection [ATCC], Rockville, MD), were cultured in RPMI 1640 medium (Invitrogen, Groningen, the Netherlands) with 10% fetal bovine serum and streptomycin. These cells were established from clear cell RCC [9]. Clear cell RCC represents 80–90% of all RCCs, and most of recent molecular-targeted drugs target clear cell RCC. About 70% of clear cell RCC features mutation or inactivation of the VHL tumor suppressor gene. As 769-P and 786-O cells have VHL mutation in each different mechanism [10], we selected the two renal cancer cell lines in our study.

### Cell viability assay

For testing sensitivity to Bisebromoamide at different concentrations (0.1, 1, and 10 μmol/L), cells were seeded in flat-bottomed 96-well plates. After 24 h, the culture medium was replaced with medium containing the reagents and then incubated for another 48 or 72 h. Cell viability was determined employing an assay for water-soluble Tetrazolium (WST)-1 salts (Takara, Shiga, Japan). At the end of the incubation period, WST reagents were added to each well and incubated for 1 h. Cell viability was estimated colorimetrically by reading color intensity in a plate reader at 570 nm. Relative viability was calculated as a percent of the control. Each experiment was performed in triplicate.

### Cell lysate preparation

Cells were placed on ice and rinsed twice with ice-cold phosphate-buffered saline, scraped off the plate, and then lysed in 100 μL ice-cold RIPA buffer (20 mmol/L tris HCl, pH 7.4, 150 mmol/L NaCl, 2 mmol/L ethylenediaminetetraacetic acid, 1% NP-40, 1% Na deoxycholate, 0.1% SDS, 50 mmol/L NaF, 1 mmol/L sodium orthovanadate, 1 mmol/L phenylmethylsulfonyl fluoride, 10 μg/mL aprotinin, and 10 μg/mL leupeptin) containing protease inhibitors. Protein concentrations in the supernatants were determined by the dye-binding method according to manufacturer's instructions (BioRad Laboratories, Hercules, CA).

### Western blotting

Fifty micrograms of total protein was separated by SDS-polyacrylamide gel electrophoresis on 12.5% acrylamide gel and transferred to nitrocellulose membranes. Nonspecific binding was blocked in tris-buffered saline containing 5% nonfat dry milk before incubation with the primary antibodies. After washing, the blots were incubated with peroxidase-labeled secondary antibody (Dako Denmark A/S, Glostrup, Denmark). Signals were detected using enhanced chemiluminescence reagents with the ECL Plus™ Western Blotting Detection System and then analyzed. Intensity was quantified using the LAS 3000 imaging system (Fujifilm, Tokyo, Japan).

### Detection of apoptosis by flow cytometry

After treatment with Bisebromoamide (1 μmol/L) for 72 h, adherent and nonadherent cells were pooled and fixed. Breaks at the 3′-OH DNA end were detected using the TUNEL technique with an ApoTag^®^ Plus Fluorescein In Situ Apoptosis Detection Kit according to supplier's instructions (Chemicon, Temecula, CA). Fluorescein isothiocyanate-labeled cells were analyzed by flow cytometry using an Epics^®^ Altra™ flow cytometer (Beckman Coulter, Fullerton, CA). The proportions of cells at different cell-cycle phases were assessed by the incorporation of BrdU/propidium iodide (PI) (Sigma) staining. After RCC cells had been exposed to the culture medium containing Bisebromoamide (1 μmol/L), the cells were stained with FITC-labeled BrdU and PI, and analyzed using a flow cytometer. Each experiment was performed in triplicate.

### Statistical analysis

All values are expressed as means ± standard error (SE). Statistical analysis was performed by Student's *t*-test with *P* < 0.05 considered significant.

## Results

### Effects of Bisebromoamide on the viability of renal cancer cells

**Figure 1:**
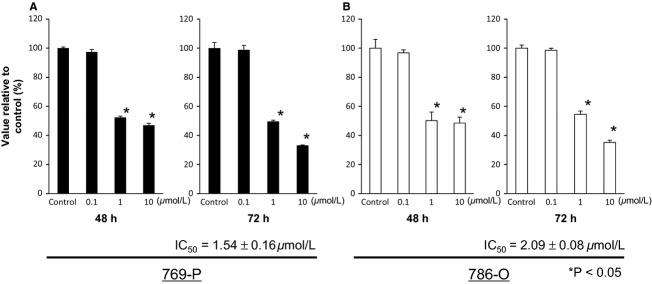
Effects of Bisebromoamide on the viability of renal cancer cells (A and B). Cells were treated with various concentrations of Bisebromoamide, and cell viability was assessed by water-soluble Tetrazolium (WST)-1 assay. Bisebromoamide dose-dependently (1–10 μmol/L) and time-dependently (48–72 h) inhibited proliferation of all cell lines. Bars represent SE. The single asterisk indicates *P* < 0.05 as compared with untreated cells. Data shown are the averages of three experiments per cell line.

After treatment with Bisebromoamide (0.1, 1, and 10 μmol/L) for 48 and 72 h, cell viability was significantly decreased in both cell lines at 1 and 10 μmol/L (Fig. 1A and B). A probit analysis of the dose–response functions showed the IC_50_ value after 72 h to be 1.54 ± 0.16 μmol/L for 769-P cells and 2.09 ± 0.08 μmol/L for 786-O cells. Based on these results, we decided to treat both cell lines with 1 μmol/L for 72 h in the following experiments.

### Induction of apoptosis by Bisebromoamide in RCC cell lines

**Figure 2:**
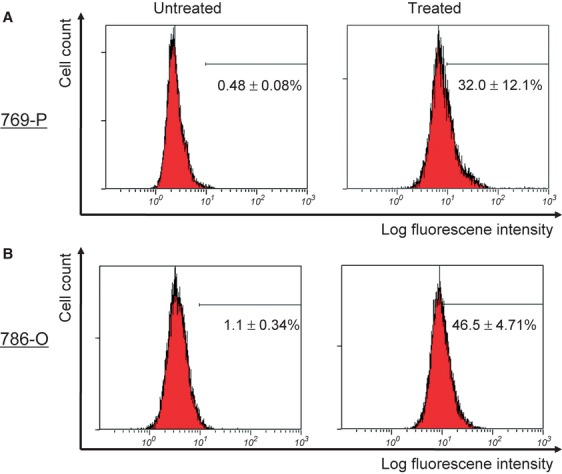
Detection of apoptosis by flow cytometric TUNEL analyses (A and B). Two renal cancer cell lines were untreated or treated with 1 μmol/L Bisebromoamide for 72 h. The cells were then stained using the TUNEL method to identify apoptotic cells. The cells with log fluorescence intensity >101 were considered positive for apoptosis.

**Figure 3:**
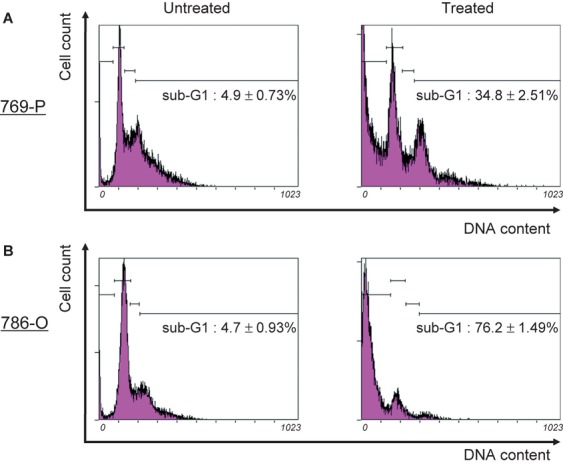
Cell-cycle analysis by flow cytometric propidium iodide analysis (A and B). A sub-G1 peak indicative of apoptosis was obtained in both cell lines treated with 1 μmol/L Bisebromoamide for 72 h.

To determine whether the treatment of cells with Bisebromoamide may lead to apoptotic cell death, flow cytometric TUNEL analysis was performed. After treatment with 1 μmol/L Bisebromoamide for 72 h, apoptosis was observed in both cell lines (Fig. 2A and B). Apoptosis was seen in only 0.48 ± 0.08% of untreated 769-P cells and 1.1 ± 0.34% of untreated 786-O cells. Bisebromoamide at 1 μmol/L for 72 h resulted in apoptosis in 32.0 ± 12.1% of 769-P cells and 46.5 ± 4.71% of 786-O cells. Furthermore, cell-cycle distributions were analyzed by flow cytometry using BrdU/PI staining. Treatment of cells with Bisebromoamide increased the percentage of cells in the sub-G1 phase (Fig. 3A and B). Expressions of cleaved caspase-3 in the two RCC lines were evaluated using Western blotting (Fig. 7A and B). After 24 h of incubation, expressions of cleaved caspase-3 were increased in both cell lines tested.

### Bisebromoamide inhibits phosphorylation of ERK, Akt, mTOR, and p70 S6

**Figure 4:**
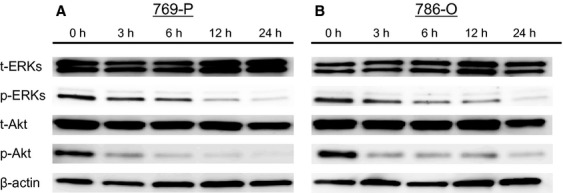
Effect of Bisebromoamide on the activation of ERKs and Akt in two renal cancer cell lines (A and B). Cells were treated with 1 μmol/L Bisebromoamide for 24 h. Changes in the expressions of ERKs and Akt were analyzed by Western blotting. Bisebromoamide downregulated the expression of p-ERKs as well as that of p-Akt in both cell lines. There were no effects on the expressions of t-ERKs and t-Akt.

**Figure 5:**
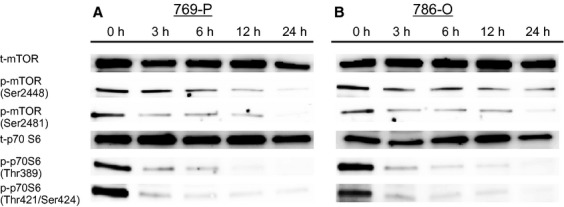
Effect of Bisebromoamide on the activations of mammalian target of Rapamycin (mTOR) and p70 S6 kinase in two renal cell carcinoma lines (A and B). Cells were treated with 1 μmol/L Bisebromoamide for 24 h. Changes in the expressions of mTOR and p70 S6 kinase were analyzed by Western blotting. Bisebromoamide downregulated the expression of p-mTOR (Ser2448 and Ser2481) as well as that of p-p70 S6 kinases (Thr389 and Thr421/Ser424), whereas there were no effects on the expressions of t-mTOR and t-p70 S6 kinase.

Expressions of t-ERKs and p-ERKs in the two RCC lines were evaluated using Western blotting. After 24 h of incubation, Bisebromoamide downregulated the expressions of p-ERKs at 1 μmol/L, while having little effect on t-ERKs in both cell lines (data not shown). Time–response experiments showed that Bisebromoamide inhibited the phosphorylation of ERK and Akt in both cell lines tested, whereas there were no evident effects on the expressions of t-ERKs and t-Akt (Fig. 4A and B). Similar effects were recognized with phosphorylation of mTOR (Ser2448 and Ser2481) and phosphorylation of p70 S6 (Thr389 and Thr421/Ser424) in both cell lines (Fig. 5A and B).

### Bisebromoamide has little effect on the upstream kinases of ERK and Akt

**Figure 6:**
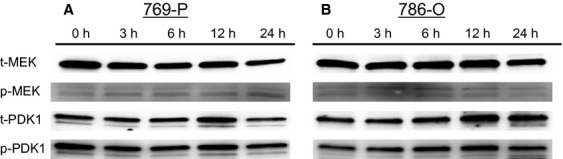
Effect of Bisebromoamide on the activations of MEK and PDK1 in two renal cell carcinoma lines (A and B). Cells were treated with 1 μmol/L Bisebromoamide for 24 h. There was little effect on the phosphorylation of MEK and PDK1 in both cell lines.

**Figure 7:**
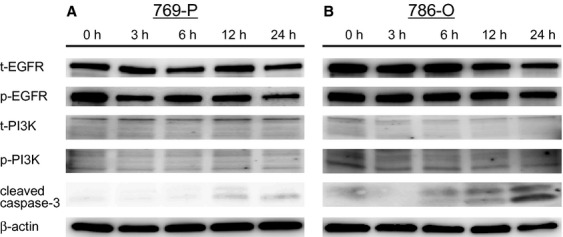
Effect of Bisebromoamide on the activations of epidermal growth factor receptor (EGFR), phosphatidylinositol 3-kinase (PI3K), and cleaved caspase-3 in two renal cell carcinoma lines (A and B). Cells were treated with 1 μmol/L Bisebromoamide for 24 h. There was little effect on the phosphorylation of EGFR and PI3K in both cell lines. Cleaved caspase-3 was upregulated after 12-h incubation in both 769-P and 786-O cells.

Expressions of MEK, PDK1, PI3K, and EGFR in the two RCC lines were evaluated using Western blotting (Figs. 6 and Figure 7A and B). Time–response experiments showed that Bisebromoamide had little effect on the phosphorylation of MEK, PDK1, PI3K, and EGFR in both cell lines tested.

## Discussion

In this study, we have demonstrated that Bisebromoamide has a cytotoxic effect on human RCC lines, and cytotoxic activity was apoptotic rather than necrotic. In addition, we showed that this compound suppressed cell proliferation in both cell lines by inhibiting the PI3K/Akt/mTOR pathway as well as the Raf/MEK/ERK pathway. These results suggest that Bisebromoamide inhibits two main signaling pathways, both of which play central roles in tumor cell proliferation. On the other hand, this drug did not inhibit the phosphorylation and expression of EGFR, MEK, PI3K and PDK1, and upstream receptors of Raf/MEK/ERK and PI3K/Akt/mTOR pathways. These results do not imply that Bisebromoamide would inhibit the expression or activation of EGFR. Rather, Bisebromoamide would inhibit the activation of ERK and Akt, not MEK, PI3K, and PDK1. Therefore, we consider that the target of Bisebromoamide would be, at least in this study, ERK and Akt in renal cancer cells.

The Raf/MEK/ERK signal transduction pathway is present in all eukaryotic cells. This pivotal pathway relays extracellular signals to the nucleus via a cascade of specific phosphorylation events involving Raf, MEK, and ERK to regulate fundamental cellular processes, including proliferation, differentiation, and cell survival [11]. The Raf/MEK/ERK pathway contributes to inducing growth factors involved in cell proliferation, as well as influencing apoptotic pathways, allowing cells to respond with the aggressive growth behavior characteristic of RCC [12]. And this pathway has important antiapoptotic effects in RCC, thereby providing an attractive target for intervention [13]. Accordingly, inhibiting this pathway might lead to induce apoptosis. Moreover, phosphorylation of ERK is an independent prognostic factor in RCC. The association between activation of mitogen-activated protein kinases (MAPKs) and the carcinogenesis of human RCCs was first reported by Oka et al. [14] in 1995. They reported that MAPK activation was detected more frequently in high-grade than in low-grade RCCs, suggesting that constitutive activation of the MAPK cascade may play an important role in the carcinogenesis of RCCs, and that increased activation of MAPK could be associated with higher malignant potential in tumors. Campbell et al. [15] showed phosphorylation of ERK to be an independent prognostic factor in RCC that was associated with advanced and aggressive pathological features and to predict the onset of metastasis in patients with localized disease. On the other hand, the PI3K/Akt/mTOR pathway regulates several normal cellular functions that are also critical for tumorigenesis, including cellular proliferation, growth, survival, and mobility. This pathway is constitutively activated in various human malignancies, including kidney, prostate, breast, thyroid cancer, and others [16], and activation of this pathway plays a critical role in tumor progression [17]. Akt, which activates the mTOR pathway, is a subfamily of the serine/threonine protein kinases and has been implicated as being crucial in controlling the balance between cell survival and apoptosis [18]. Horiguchi et al. [19] demonstrated elevated activation of Akt to have a significant association with higher grade metastatic disease and poor survival in RCC. These results support our observation that Bisebromoamide induces apoptosis of RCC lines by inhibiting phosphorylation of both ERK and Akt.

Bisebromoamide is a novel cytotoxic peptide which was shown to specifically inhibit the phosphorylation of ERK. However, in this study, we demonstrated that this new drug acts as a multitarget kinase inhibitor that may directly block tumor growth by inhibiting several other kinases such as ERK, Akt, and mTOR in human RCC lines.

Effective inhibitors specific for many of the key components of the Raf/MEK/ERK or PI3K/Akt/mTOR pathway have been developed. For example, CI-1040, an oral MEK inhibitor, shows antitumor activity in patients with advanced non–small-cell lung, breast, colon, and pancreatic cancer [20]. Sorafenib, or BAY 43-9006, which targets Raf/MEK/ERK pathway, produced significant tumor growth inhibition and a reduction in 786-O tumors [21]. And this inhibition of angiogenesis correlated with increased level of tumor apoptosis. The oral kinase inhibitor of Raf is already in widespread clinical use for patients with advanced RCC [5]. NVP-BEZ235, which targets PI3K/Akt/mTOR pathway, induced growth arrest in 786-O that was associated with inhibition of Akt and S6 phosphorylation as well as the induction of apoptosis and reduction in markers of tumor cell proliferation [2]. This drug is an inhibitor of PI3K and mTOR kinase activity, which effectively and specifically blocks dysfunctional activation of the PI3K pathway, inducing G1 arrest [1]. Everolimus (RAD001) and Temsirolimus (CCI-779) are inhibitors of mTOR, and both are already in widespread clinical use for patients with advanced RCC [3,4]. Thus, specific inhibitors have been devised and some are currently in clinical trials or already in use. However, none of these drugs inhibits more than one pathway.

Although VEGF and mTOR inhibitions have dramatically improved outcomes for patients with metastatic RCCs, the goal of cure remains elusive. Our findings have important implications for metastatic RCC therapy because they provide a rationale for dual inhibition of the Raf/MEK/ERK and PI3K/Akt/mTOR pathways. Previous reports indicated the efficacy of combining Raf/MEK/ERK inhibitors with PI3K/Akt/mTOR inhibitors. Carracedo et al. [22,23] demonstrated that mTORC1 inhibition leads to Akt activation through upregulation of receptor tyrosine kinases, and the combination of mTORC1 and MAPK inhibitors enhanced the growth inhibitory effect of rapamycin in several human cancer cell lines. Furthermore, Roulin et al. reported that combining NVP-BEZ235 with sorafenib increased antitumor efficacy as compared with either drug alone in RCC lines. To our knowledge, there are no previously reported drugs that inhibit both the Raf/MEK/ERK and the PI3K/Akt/mTOR pathways, which can be administered as a single agent in human RCC. In conclusion, Bisebromoamide suppresses RCC proliferation and potentiates apoptosis by inhibiting both the Raf/MEK/ERK and the PI3K/Akt/mTOR pathways. Bisebromoamide may thus be a promising agent for the treatment of RCC.
